# Volumizing threads and hyaluronic acid filler for lip augmentation

**DOI:** 10.1111/srt.13797

**Published:** 2024-06-17

**Authors:** Jovian Wan, Lisa Kwin Wah Chan, Kar Wai Alvin Lee, Hugues Cartier, Kyu‐Ho Yi

**Affiliations:** ^1^ Asia‐Pacific Aesthetic Academy Hong Kong Hong Kong; ^2^ EverKeen Medical Centre Hong Kong Hong Kong; ^3^ Centre Médical Saint Jean Arras France; ^4^ Division in Anatomy and Developmental Biology, Department of Oral Biology Human Identification Research Institute, BK21 FOUR Project, Yonsei University College of Dentistry Seoul South Korea; ^5^ Maylin Clinic (Apgujeong) Seoul South Korea

Dear Editor,

The lips have long been recognized as a defining feature of youthfulness, attractiveness, and beauty. Lip augmentation procedures are popular in aesthetic practice, aimed at achieving defined and full lips. While hyaluronic acid fillers are commonly used for lip augmentation, their dynamic nature may lead to accelerated breakdown, necessitating additional measures to maintain results.^1^, ^2^, ^3^, ^4^, ^5^, ^6^ Volumizing threads, though relatively new in the literature, offer promise as an adjunct to fillers for lip augmentation. We present a successful case of lip augmentation using volumizing polydioxanone (PDO) threads, emphasizing significant lip volumization. Moreover, the potential benefits of PDO threads, such as collagen stimulation and minimal downtime, are delineated. Further research, including randomized controlled trials, is imperative to evaluate the long‐term safety and efficacy of PDO thread procedures for lip augmentation.

Throughout history, the contour of the lips historically has consistently played a significant role in defining overall youthfulness, attractiveness, and beauty.^7^, ^8^ Therefore, demands for lip augmentation are prevalent in aesthetic practice.^9^ Defined and filled lips are associated with allure and aesthetic appeal. Factors including reduced volume and elasticity of the underlying soft tissue, recession of the vermilion border, bone resorption, dental loss, smoking, and sun exposure collectively contribute to the aging process in the perioral region, potentially impacting its aesthetic appeal. Moreover, lips are vulnerable to environmental irritants, which can compromise the integrity of their delicate outer layer, further accentuating signs of aging and reducing perceived attractiveness.^10^, ^11^ Hyaluronic acid fillers are commonly used for minimally invasive lip augmentation procedures.^9^ However, the dynamic nature of the lips often accelerates the breakdown of filler material, necessitating additional measures to maintain results.^12^ Consequently, volumizing threads are often incorporated in combination with fillers to create a scaffold effect. While standalone thread application may present challenges in shaping the lips, practitioners commonly adopt a combination therapy approach.^13^ Nonetheless, when specifically defining the vermilion border, threads can be employed independently. The decision regarding thread placement, whether targeting the vermilion border or focusing on volumization, is typically made during the insertion process, with insertion points located approximately 1.5 cm laterally from the modiolus or cheilion's lateral border.

There is currently limited literature available on the topic of lip augmentation using volumizing threads. Our objective is to address this gap by presenting a successful case of lip augmentation utilizing volumizing threads. Additionally, we aim to provide further insight into the procedure by demonstrating the thread insertion technique through a supplementary video.

A 35‐year‐old lady presented at the clinic seeking treatment for thin lips. She had previously undergone lip augmentation with hyaluronic acid fillers 3 years ago. Additionally, she received monthly laser therapy for facial contouring and filler injections to the nasolabial folds 1 year ago. There was no significant medical history of note. The procedure involved inserting threads into the submucosal layer of the lips. Four 21‐gauge 5 cm N‐Scaffold threads were inserted, with two on each side of the upper and lower lips (refer to Video 1 and Figure 1). Furthermore, 1 cc of hyaluronic acid filler was administered. The patient attended biweekly follow‐up appointments for 1‐month post‐thread insertion, during which no adverse events or complications were noted. A follow‐up photograph was taken at the 1‐month mark to evaluate the outcomes (Figure 2). The patient expressed high satisfaction with the results, noting that while the augmentation appeared subtle, the volumization felt significantly improved.

**FIGURE 1:**
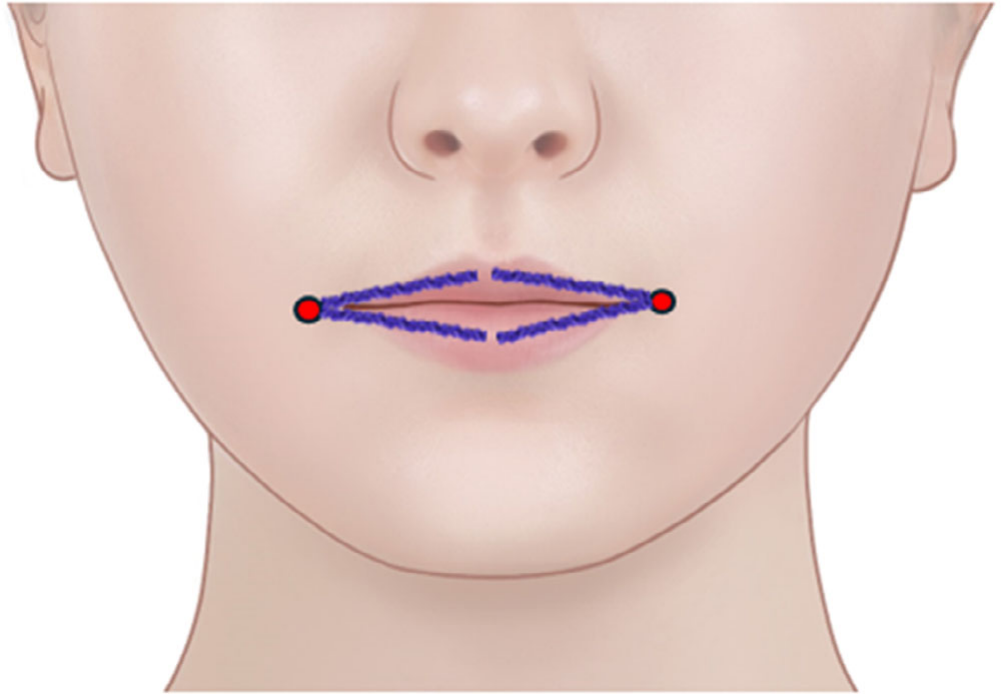
A total of four 21‐gauge 5 cm N‐Scaffold threads were inserted, with two on each side in lower and upper lips. The red dot is the entry point located 1.5 cm to the cheilion.

**FIGURE 2:**
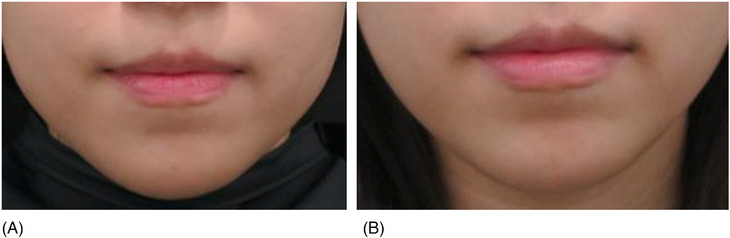
A 35‐year‐old woman received treatment with volumizing threads, opting for the use of 21G volumising threads (N‐scaffold, Nfinders, Korea). Four lines of threads were individually inserted into each of the left and right, as well as the lower and upper lips. Additionally, 1 cc of hyaluronic acid filler was injected for enhancement. Photograph (A) was taken before the treatment, and photograph (B) depicts the results 1‐month after the treatment.

Lip augmentation is a frequently performed procedure in aesthetic medicine, often employing hyaluronic acid fillers as the preferred choice among practitioners. This preference is attributed to the versatility, biocompatibility, and reversibility of hyaluronic acid fillers, making them the cornerstone of lip augmentation treatments.^9^ However, there are significant safety concerns associated with injecting fillers into the lips, particularly regarding vascular occlusion. Accidental injection into blood vessels can lead to skin necrosis and pose a low risk of blindness if the filler travels and blocks the central retinal artery.^14^, ^15^

In aesthetic medicine, PDO is frequently utilized for facial rejuvenation and body contouring, wherein common complications like bruising, bleeding, and temporary redness are typically minor and short‐lived.^16^, ^17^, ^18^, ^19^, ^20^ Lip augmentation with PDO threads, although relatively new and not extensively documented in literature, offers various benefits. These include stimulation of collagen production, immediate results, minimal downtime compared to surgical procedures, versatility in addressing lip augmentation, defining the vermilion border, and managing perioral rhytids.^21^, ^22^ Additionally, PDO threads can be combined effectively with hyaluronic acid filler for enhanced outcomes.^13^

A study by Horn et al.^23^ introduced a novel approach to treat gummy smiles by utilizing polyester threads as a physical barrier to impede muscle reinsertion. The choice of polyester threads as the filling material was based on their favorable characteristic, including compatibility with oral tissues, ensuring integration into the surrounding environment. Additionally, these threads prompt the formation of a fibrous tissue capsule upon insertion, aiding in the prevention of muscle reattachment.^24^ The study's clinical outcomes showcased the effectiveness of this technique in correcting gummy smiles, with patients reporting high aesthetic satisfaction and biological stability. Although this study presents the use of polyester threads for treating gummy smiles, which differs from the material and indication in our case, it serves as a pertinent example of thread application in the perioral region.

In conclusion, our case report demonstrates the efficacy of lip augmentation using volumizing PDO threads as a safe and effective procedure. The patient's satisfaction with the results underscores the success of this intervention in achieving desired aesthetic outcomes. Further research, including randomized controlled trials, is needed to evaluate long‐term safety and efficacy in PDO thread procedures tailored for lip augmentation.

## CONFLICT OF INTEREST STATEMENT

The authors declare no conflicts of interest.

## Data Availability

The data are available by contacting to corresponding author.
